# Raman microspectroscopy: sub-cellular chemical imaging of aging

**DOI:** 10.18632/aging.203785

**Published:** 2021-12-13

**Authors:** Lisa Liendl, Markus Schosserer

**Affiliations:** 1 Institute of Molecular Biotechnology, Department of Biotechnology, University of Natural Resources and Life Sciences, Vienna, Austria; 2 Christian Doppler Laboratory for Skin Multimodal Imaging of Aging and Senescence, Vienna, Austria; 3 Austrian Cluster for Tissue Regeneration, Vienna, Austria

**Keywords:** aging, cellular senescence, Raman microspectroscopy, correlated multimodal imaging, chemical imaging

Aging is well known to represent a heterogeneous process at the species, individual, and tissue levels. However, accumulating evidence further suggests significant variability even at the cellular and sub-cellular scales. Unfortunately, most current methods still measure manifestations of aging only in whole organisms and tissues, or in cells *in vitro*. Together with modern single-cell sequencing, mass cytometry, and histological staining technologies, chemical imaging represents, in our view, an essential addition to the state-of-the-art toolbox to analyze the heterogeneity of aging at sub-tissue resolution.

Chemical imaging describes the combination of a microscope with a chemical analyzer, such as a Raman-, infrared-, or mass-spectrometer. This setup permits the chemical investigation of small objects down to sub-cellular resolution, whereby different methods provide different spatial resolution, invasiveness, and density of chemical information.

Raman microspectroscopy is exceptionally promising for biological samples because it can be performed label-free and in a non-invasive manner, offering a wide range of applications and bringing new possibilities to aging research, recently reviewed by us [1]. This methodology exploits vibrational modes occurring due to the interaction of light and polarizable molecules in the material to be analyzed. The resulting Raman scattering, also called inelastic scattering, is a rare phenomenon compared to elastic or Rayleigh scattering and can only be taken advantage of due to highly sophisticated and sensitive instrumentation [2]

Especially skin aging has been the subject of Raman-based *in vivo* investigations, seeking spectral regions suitable for classifying young, chronologically aged, and photoaged skin. Apart from alterations in prominent spectral regions resembling diverse biological macromolecules at the cellular level, changes in water content seem to be reflected in Raman spectra and might correlate with age groups. A recent study further analyzed water content in different stratum corneum layers, revealing similar amounts at the surface. At the same time, the authors found higher water content in deep parts of the stratum corneum and viable epidermis in aged skin [3]. This is explained by an age-dependent change in the amount of bound and unbound water. Furthermore, they observed a reduced lipid to protein ratio in the skin from older persons. Raman signatures of other tissues, including blood, eyes, bones, and teeth, were already analyzed to understand aging-related changes [1].

The detection and comprehensive characterization of senescent cells *in vivo* still pose a challenge for the efficacy testing of senotherapies, one of the most promising current therapeutic strategies to mitigate or revert aging-related pathologies. Importantly, senescent cells were already successfully distinguished from non-senescent cells via their Raman signatures. Eberhardt and colleagues analyzed human dermal fibroblasts in 2D cell culture and 3D structures of fibroblast-derived matrices [4,5]. Differences in spectra from senescent and proliferating cells could be assigned to main spectral regions, including amide I, II, and III, referring to protein contribution and nucleic acid and lipid-based alterations. Our group demonstrated that supernatants from artificial skin equivalents were sufficient to distinguish organoids containing senescent dermal fibroblasts from those without senescent cells [6].

The complex biochemical composition of cells and tissues implies that the analysis of corresponding Raman spectra is anything but simple. Peaks and spectral bands can be assigned to chemical features; however, all biomolecules are similarly structured and share the same chemical bonds. Thus, assigning a peak to a specific class of biomolecules and even further to a particular protein or lipid, for example, is challenging and often impossible. In many cases, complex multivariate statistical analysis is required to decipher subtle spectral changes. The interpretation of spectra is challenging and must be conducted carefully to extract meaningful biological information while avoiding overinterpretation. Another challenge represents the dependence of the spectra on the respective Raman microscope configuration and the differences in instrumentation across laboratories. More studies are needed to understand the resulting spectral variations and to develop strategies for correcting them [7]. Finally, acquiring high-resolution Raman maps is still time-consuming because Raman-scattered photons are rare, and therefore, long integration times are usually required. Using sensitive detectors and powerful lasers can partially circumvent this problem. Nonetheless, it can take up to several hours to map a single cell at a high spatial resolution and reasonable signal/noise ratio, thus often limiting this method to fixed tissues.

Nonetheless, mapping approaches often constitute an advantage as they result in multidimensional data containing spectral and spatial information, which can directly be assigned to morphologic features. The true power of Raman microspectroscopy is unleashed when Raman maps are aligned with the visual and chemical information gathered from other imaging modalities. This correlated multimodal imaging approach is not limited to light, fluorescence, and Atomic Force Microscopy (AFM), with which current Raman microscopes are often coupled. The possibilities to combine different imaging modalities, including Raman-, infrared-, imaging mass spectrometry, confocal microscopy, electron microscopy, and super-resolution fluorescence microscopes are endless [8]. Identifying suitable multimodal imaging pipelines, consisting of compatible sample preparation and imaging steps, as well as bioinformatics to combine and register different data types, is often challenging. Still, in the future, these methods might provide a more detailed understanding of biological aging processes at sub-tissue or sub-cellular resolution.
